# Effectiveness of 6 Months of Tailored Text Message Reminders for Obese Male Participants in a Worksite Weight Loss Program: Randomized Controlled Trial

**DOI:** 10.2196/mhealth.3949

**Published:** 2015-02-03

**Authors:** Ju-Young Kim, Sohee Oh, Steven Steinhubl, Sohye Kim, Woo Kyung Bae, Jong Soo Han, Jeong-Hyun Kim, Keehyuck Lee, Mi Jin Kim

**Affiliations:** ^1^Department of Family MedicineSeoul National University Bundang HospitalSeongnam-siRepublic Of Korea; ^2^Department of BiostatisticsSeoul Metropolitan Government Seoul National University Boramae Medical CenterSeoulRepublic Of Korea; ^3^Scripps Translational Science InstituteDepartment of Digital MedicineLa Jolla, CAUnited States; ^4^Department of Medical NutritionGraduate School of East-West Medical Science Kyung Hee UniversityYongin-siRepublic Of Korea; ^5^Health Promotion CenterDepartment of Family Medicine,Seoul National University Bundang HospitalSeongnam-siRepublic Of Korea; ^6^Health Promotion CenterDepartment of Famliy MedicineSeoul National University Bundang HospitalSeongnam-siRepublic Of Korea; ^7^Department of NeuropsychiatrySeoul National University Bundang HospitalSeongnam-siRepublic Of Korea

**Keywords:** weight reduction program, text messaging, worksite, health promotion

## Abstract

**Background:**

Worksite nutrition and physical activity interventions are important to help overweight and obese employees lose weight, but costs and insufficient sustained motivation prevent the majority of these programs from succeeding. Tailored text messaging in aiding weight management has been effective in several studies, but no studies have evaluated the effect of a tailored text message service on weight loss in a worksite health promotion program.

**Objective:**

We studied the efficacy of a tailored text-messaging intervention for obese male participants in a worksite weight loss program of 6 months duration.

**Methods:**

The study was an unblinded, randomized controlled trial. Men with a body mass index greater than 25 kg/m^2^ were recruited from the Korea District Heating Corporation, the Korea Expressway Corporation, and the Korea Gas Corporation. The participants were identified by nurse managers. Participants were randomly allocated to 1 of the following 2 groups for 24 weeks: (1) intervention group, which received tailored text message reminders every other day plus 4 offline education sessions and brief counseling with monthly weight check by nurses for weight control over 6 months and (2) control group, which received the 4 offline education sessions and brief counseling with monthly weight check by nurses about weight control over 6 months. The primary outcome was the difference in weight loss at 6 months. A mixed-model repeated-measures analysis was performed to evaluate the effect of the intervention group’s weight loss compared with the control group.

**Results:**

A total of 205 obese men were randomized into either the intervention (n=104) or the control group (n=101). At the end of 6 months, the intervention group (n=63) had lost 1.71 kg (95% CI –2.53 to –0.88) and the control group (n=59) had lost 1.56 kg (95% CI –2.45 to –0.66); the difference between the 2 groups was not significant (mean difference –0.15, 95% CI –1.36 to 1.07). At the end of the study, 60% (34/57) of the intervention group rated the message program as helpful for weight control and 46% (26/57) would recommend the text message service to their friends.

**Conclusions:**

Tailored text message reminders did not have a significant effect on weight loss in obese men as part of a worksite weight loss program.

**Trial Registration:**

International Standard Randomized Controlled Trial Number (ISRCTN): 39629189; http://www.isrctn.com/ISRCTN39629189?q=39629189&filters=&sort=&offset=1&totalResults=1&page=1&pageSize=10&searchType=basic-search (Archived by WebCite at http://www.webcitation.org/6VsFkwJH6).

## Introduction

Obesity is an important and modifiable risk factor for many of the leading causes of death worldwide, such as myocardial infarction, stroke, and obesity-related cancers [1,2]. Obesity also has negative effects on work performance and increases absenteeism [3,4]. Worksite weight management [5] is important for educating employees about the benefits of maintaining a healthy, sustainable weight and in helping to influence behavior, such as by encouraging healthier food choices and physical activity. Worksite weight management programs also support behavior change over time by establishing a more consistent and healthy environment. Retention [6] within worksite programs has been found to be twice that observed in commercial weight loss programs, improving long-term weight management [7].

The use of text messaging in health behavior change [8-10] has been adopted more and more frequently, not only because of its wide availability, low cost, and instant effects on users, but also because of its effectiveness in encouraging health behavior changes.

As a rule, developing and implementing worksite weight management programs requires sufficient resources, including funding, time, staff, and space. Additionally, there are reported barriers to participation [11,12] in worksite programs, including insufficient incentives, inconvenient locations, and time limitations.

Using text messages in worksite weight management programs can be useful for overcoming the time, location, and cost barriers. Some studies have used telephone coaching [13] or online weight management programs [14] in worksite weight management programs, but no studies have reported on the effects of text messaging on weight loss in worksite weight management programs. Therefore, we conducted a 6-month randomized controlled trial to determine whether a tailored text-messaging reminder service would be more effective in encouraging weight loss compared with the standard care in worksite weight management programs.

## Methods

### Recruitment

The study population consisted of employees of public institutions undergoing standardized annual medical examinations at local cooperative hospitals. The standardized medical examinations include evaluating lifestyle risk factors, such as smoking, drinking, and lack of exercise; anthropometric measurements, such as weight, height, and blood pressure; and laboratory tests, such as fasting glucose and lipid panel. Nurses managed worksite disease prevention efforts in the health promotion programs at these public institutions.

The World Health Organization (WHO) Regional Office for the Asia Pacific Region recommends defining obesity in Asians as those with a body mass index (BMI) ≥25 kg/m^2^ [15]. The Korean Society for the Study of Obesity also studied the cutoff of BMI for obesity-related disease [16] and adopted the definition. Now Korean government organizations officially use this definition when defining and implementing health policies regarding obesity in Korea.

Participants were recruited in 2011 from 3 public institutions with the help of managing nurses: the Korea Gas Corporation (KOGAS), the Korea District Heating Corporation (KDHC), and the Korea Expressway Corporation (KEC). KOGAS was incorporated by the Korean government in 1983 and has grown to become the world’s largest liquefied natural gas importer with 3437 employees working as of January 2014. KDHC was established in 1985 and 1345 people are employed in the district cooling and heating business, community energy system business, electricity business, and the new, renewable energy business. KEC was established in 1969 and has contributed to a speedy, convenient, safe transport service by constructing and maintaining expressways with a total of 4484 employees working. All headquarters of these companies are located in Seongnam and all potential participants in our study were working at headquarters of these companies mostly as teams within the administrative, management, or research departments.

The nurses screened potential participants for the following inclusion criteria: age between 20 and 60 years, obesity determined by a BMI >25, not taking medications known to cause weight gain, owning a mobile phone, and using text-messaging services. Most employees of KOGAS, KDHC, and KEC are male. In particular, more than 98% (217/222) of the employees who were eligible for this study were male. Only 5 females wanted to participate in our program and 4 were in their twenties and had no significant metabolic risks; therefore, we decided to include only males in the final study. Finally, 80 male employees of KOGAS, 81 of KDHC, and 44 of KEC agreed to participate in our study. The Institutional Review Board of Seoul National University Bundang Hospital approved this study (IRB number: B-0908-082-014).

### Intervention Group

The study evaluated a text message-based application that was tailored to participants’ individual dietary behaviors and physical activity levels using responses to questionnaires and metabolic risk profiles that were assessed by laboratory examinations and anthropometric measurements. We assessed total calorie intake with relative proportions of macronutrients such as carbohydrate, protein, and fat, sodium intake per day, as well as eating patterns such as regularity of meals, night eating, and frequent snack intake during an education session using self-administered 24-hour dietary recall [17]. The 24-hour dietary recall was collected by self-administered methods instead of being evaluated by trained interviewers and was only used for development of content of tailored text-messaging program and not for outcome evaluation.

Eating behaviors such as emotional eating, social influences, convenience food, and alcohol consumption by frequency and amount were also assessed for the purpose of the text-messaging program.

Physical activity level was assessed and categorized using the International Physical Activity Questionnaire-Short Form [18] (IPAQ-SF).

Fasting glucose, uric acid, and serum triglyceride levels were also categorized to aid in the development of tailored text messages. The contents of the text messages were developed to be both automatic and personally tailored to participants’ lifestyles, eating pattern with behaviors, and metabolic risk factors, and the messages were unidirectional. Three family physicians, 1 psychiatrist, and 2 dietitians collaborated to develop the text message contents with regard to motivation, nutritional tips, helpful recipes based on individual risk factors, and exercise tips with motivation. Text messages were sent to the intervention group 3 times a week in the morning and consisted of “goal setting and behavior change,” “education and tips for nutrition,” and “exercise and get more active” themes.

Goal setting and behavior change included reminders of the target goal set by participant and motivational messages for encouraging self-monitoring of their weight, the need for weight control, overcoming barriers, and reducing emotional eating. We especially subcategorized first attempters and history of fluctuating weight loss cycles, or yo-yo dieters, and sent tailored motivational messages to those 2 groups. For first attempters, messages were sent to start and stick to their plan, but for yo-yo dieters messages were focusing on how to prevent weight cycling again. Education and tips for nutrition included general information such as meal replacements and substitutions, meal planning, tips for eating out, and tailored information according to self-administered 24-hour dietary recalls with questionnaires. If a participant got more than 60% of energy by carbohydrate intake or 20% of energy by fat intake, messages were sent to inform them of the ideal proportion of macronutrients and to recommend healthy alternative choices. If a participant was drinking more than 35 g/day of alcohol by the frequency and amount of drinking over the past week, then he would get reminder messages regarding risk of alcohol with weight, education about empty calories, and suggestions of safe drinking amount. We also categorized tailored text messages by laboratory results based on health examination data. We applied the criteria of metabolic syndrome by the National Cholesterol Education program Adult Treatment Panel lll [19] for tailored text message contents. If systolic blood pressure was more than 135 mm Hg or diastolic blood pressure was more than 85 mm Hg or participants were on hypertension treatment, the participant received tailored messages about nutritional tips for lowering blood pressure [20] from randomized clinical trials. We classified as high triglyceride group when a participant had a triglyceride level more than 150 mg/dL or was on medication for dyslipidemia, and as prediabetic group or diabetic group if the glucose level was more than 100 mg/dL or participant was on antidiabetic medication.

For exercise and get more active, we categorized physical activity levels according to guideline of IPAQ-SF and personalized messages were sent according to low, moderate, and high activity levels. We also sent general tips for increasing nonexercise physical activity; education on the type, duration, and frequency of exercise; and showing the calories burned per exercise type per hour. Examples of the text messages’ contents are shown in Table 1.

Because we generated the content of text messages according to the results of a routine annual health examination, study participants were not reclassified in terms of types of text messages received during the study period.

Our research group visited the worksites and provided educational group sessions with printed materials on managing obesity and feedback based on self-reported questionnaires at baseline, 1 month, 3 months, and 6 months. At baseline visits, participants were told about obesity-related comorbidities, the importance of healthy weight, methods of losing weight by choosing healthy low calorie foods with examples of 1500 to 1800 low calorie meal plans, and a brief explanation of the purpose and methods of our study again. At the 1-month visits, a detailed explanation of types and frequencies of exercise for losing weight was given with videos. Also, methods for increasing nonexercise physical activities and healthy carbohydrates, such as whole grain or high-fiber foods, were given to participants. At the 3-month visits, we gave a lecture regarding stress-related eating and how to cope with that problem. We also gave nutritional information regarding healthy fat and adequate protein intake to participants. At the final visits, we focused on maintenance of weight loss (eg, problems with regaining weight) and how to prevent them. After the education session, participants were required to fill in brief questionnaires such as the IPAQ-SF and measure their weight and body fat. The printed materials were different at each educational session according to the topic. Each session was held for 40 minutes with 20 to 30 attendees. For each worksite, a total of 6-8 educational sessions took place during the 6-month study period depending on available numbers of participants. For each individual who completed the study, total educational sessions would be 4 maximum.

Nurses checked weights and offered brief counseling about diet, exercise, coping with emotional eating, tips for avoiding overeating, weight maintenance strategies, and encouraged participation in the program every month.

We used a commercial automated text message sender (Munjanara, Seoul, Korea) [21] for the tailored messages, and we could not provide interactive feedback or track and show cumulative weight changes.

**Table 1:** Text message contents.

Category subcategories classification			Example of content	Frequency
**Goal setting and behavior change**				
	**Goals**			Once a week rotating
		Target weight reminder	Remember, your goal is 77 kg in 6 months. Imagine your healthy body and get started!	
	**Motivation**			Once a week rotating
		First attempt	You just started a great jump in your healthier life. Congratulations!	
		History of yo-yo dieting	Weight cycling can be a normal process in weight loss, but you need to keep track of your weight.	
**Education and tips for nutrition**				
	**Eating behavior**			Once a week rotating
		Rapid eating	Take time to eat your meals and save yourself from future health problems.	
		Irregular eating	Skipping your meal can lead to overeating at the next meal.	
		Emotional eating	If you are upset or frustrated, stop and think for a moment, and then choose your action.	
	**Alcohol consumption** ^a^			Once a week rotating
		Problem drinking	Problem drinking can put you in danger in both body and mind.	
	**Increased blood pressure**			Once a week rotating
		SBP ≥130 mm Hg or DBP ≥85 mm Hg	Make your meal with colorful vegetables and protein-rich foods.	
	**Dyslipidemia**			
		TG ≥150 mg/dL	Diets high in refined carbohydrates and heavy alcohol use can increase triglycerides.	Once a week rotating
	**Impaired fasting glucose**			Once a week rotating
		FPG ≥100 mg/mL	You are at high risk for diabetes. Eating nutritious, balanced meals with regular exercise can protect you.	
**Exercise and get more active**				
	**Level of physical activity** ^b^			Once a week
		Low	Move yourself for just 30 minutes a day, and you have your healthy body shape.	
		Moderate	If you want to lose weight, you need to add vigorous-intensity activity at least 75 minutes per week.	
		High	You are doing great, but be careful of your joints.	

^a^ At-risk drinking: assessed by the frequency and amount of drinking over the last week and categorized as high-risk intake if participants had an average daily consumption of >35 g/day.

^b^ Level of physical activity: assessed with the International Physical Activity Questionnaire-Short Form (IPAQ-SF) and categorized as follows: low: Individual does not meet the criteria for category 2 or 3; moderate: 5 or more days of any combination of walking, moderate-intensity, or vigorous-intensity activity to achieve a minimum total physical activity level of at least 600 MET-minutes/week; high: vigorous-intensity activity on at least 3 days to achieve a minimum total physical activity of at least 1500 MET-minutes/week or 7 or more days of any combination of walking, moderate-intensity, or vigorous-intensity activities achieving a minimum total physical activity level of at least 3000 MET-minutes/week.

### Comparison Group

The comparison group received the same educational group sessions with printed materials at baseline, 1 month, 3 months, and 6 months, and their weights with percent body fat were checked. Also, questionnaires regarding physical activities were evaluated and they got brief counseling about diet, exercise, coping with emotional eating, tips for avoiding overeating, and weight maintenance strategies by nurses at their offices. The comparison group received identical support as the intervention group with the exception of not receiving automatic tailored text messages.

### Study Design

Individuals eligible for this study were invited to participate in the program by the study coordinator after they completed their annual health examinations at the hospital. Participants were informed about the text-messaging intervention at their worksites’ weight reduction education programs and gave written informed consent to use their hospital information in the text messages. Participants were asked to contact the researchers by telephone or email if they had questions regarding the trial.

Evaluation of the text-messaging program was based on an unblinded, randomized controlled trial with a 1:1 match of the intervention group to the comparison group. Random allocation was performed by blocked randomization, with block sizes of 4 and 6, and using Web-based randomization at the Medical Research Collaborating Center of Seoul National University Hospital. A trained research assistant who did not participate in running the educational sessions or in recruiting potential participants scheduled and sent the text messages to the intervention group using the commercial automatic text message sender. There were no methodological changes during the study period.

**Figure 1:**
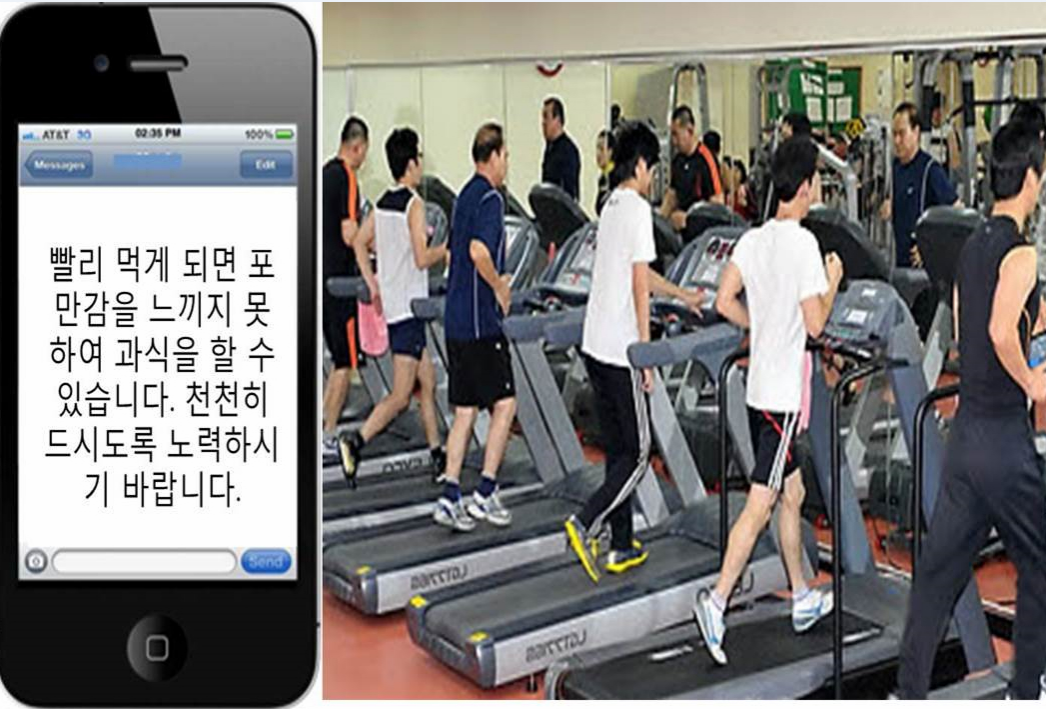
Screenshot of text message reminders in worksite weight loss program.

Both groups received the equivalent of US $10 in reimbursement at each educational session. The study took place between May and December 2011 in Seongnam, Korea. A screenshot of this study app is shown in Figure 1.

### Measurement

#### Weight and Percent Body Fat

Weight change was the primary outcome and was evaluated with percent body fat at baseline, 1 month, 3 months, and 6 months, and measured by nurses using portable bioelectrical impedance analysis (InBody U20, Seoul, Korea).

#### Physical Activity Questionnaires

Physical activity was assessed using the Korean version of IPAQ-SF [18] at baseline, 1 month, 3 months, and 6 months.

#### Obesity-Related Quality of Life

We also evaluated the Korean versions of validated obesity-related quality-of-life scales [22] at baseline and 6 months. These were self-administered questionnaires to measure obesity-related quality of life.

#### Satisfaction and Acceptability

Satisfaction with and acceptance of this text message program in the intervention group were evaluated at 6 months by rating the overall program (eg, Were you satisfied with the text message program? How much did the text messages help you with weight management?) with a 5-point Likert rating (from strongly agree to strongly disagree). “Somewhat agree” and “strongly agree” were considered positive responses.

#### Eating Behavior

We evaluated eating behavior based on eating speed, irregular eating, and emotional eating at baseline to develop the content of the tailored text messages, but we did not use eating behavior for outcome measurement. We also evaluated baseline alcohol intake as the frequency and amount of drinking over the last week and categorized intake as high risk if participants had an average daily consumption of more than 35 g/day based on the risk of metabolic syndrome [23]. Alcohol consumption was only used for the content of the text messages, not for outcome evaluation.

### Statistical Analysis

We calculated sample size to detect a group difference of 1.65 kg at 6 months based on a previous study [24], which was a mean 1.72 kg (SD 3.3) difference in changes of body weight at 4 months between intervention and control groups. A total of 124 participants provided a power of 0.8 with alpha=.05. Allowing for an attrition rate of 30%, a total of 207 participants were needed to perform this study. Differences in the baseline categorical variables between groups were analyzed using chi-square tests and continuous variables were compared using *t* tests. Group differences in the effects of the text-messaging intervention on weight changes were analyzed using a linear mixed-effects model for repeated measures [25], which used all available data and provided valid results in the event of missing data (which were under the missing at random mechanism). The model considered age, treatment group, time, and group by time interactions as fixed effects and incorporated random effects for individual participants to consider variabilities in individuals across time. As we weighed study participants with the same portable bioelectrical impedance measurement device, we also evaluated the change of percent body fat.

Physical activity levels and obesity-related quality-of-life scores were also analyzed with a linear mixed model. All analyses were performed using SAS 9.3 (SAS Institute, Cary, NC, USA) and R version 2.15.2 [26] software. *P* values less than .05 were considered statistically significant.

## Results

### Enrollment and Retention

**Figure 2:**
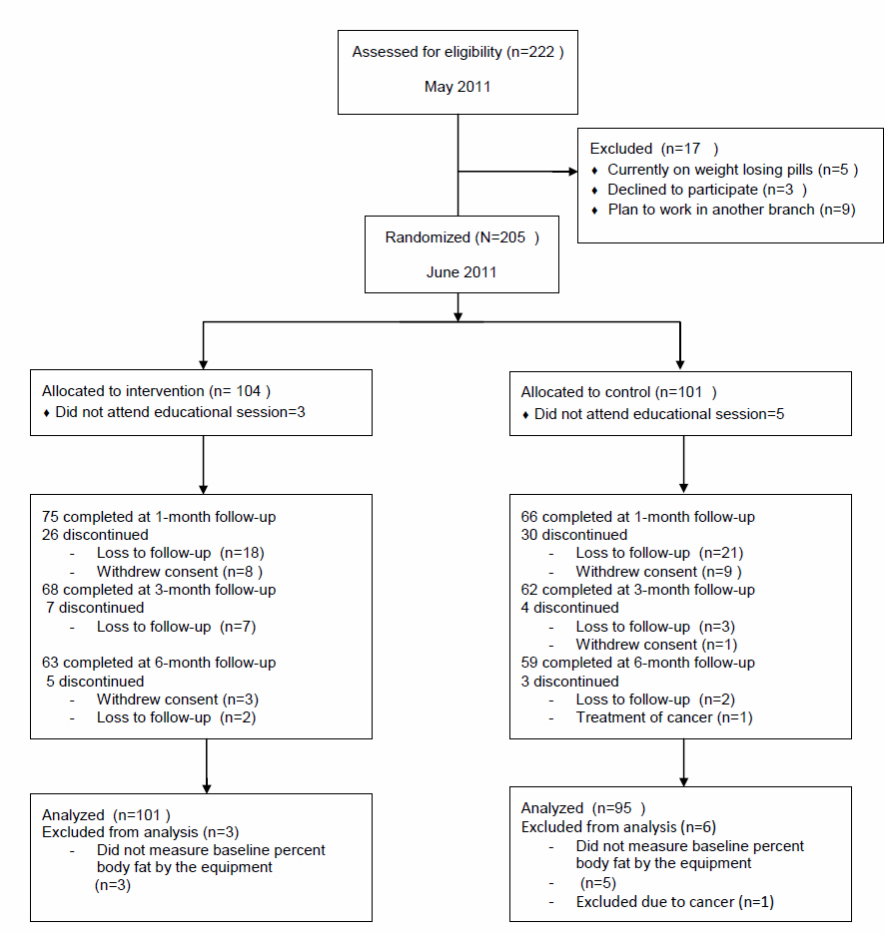
Flow diagram for participant enrollment and retention.

Study enrollment and retention flow are shown in Figure 2. A total of 222 individuals were eligible for participation as assessed by the managing nurses. Among those eligible, 205 agreed to participate and comprised the final study population and were randomized to either the text message group (n=104) or the control group (n=101). Baseline weight and body fat percentage were measured at the educational sessions with portable body impedance analysis; if a participant did not attend the session, we could not obtain his baseline body weight. Because 9 individuals did not attend the baseline session, the final analysis included 101 men from the text message group and 95 from the control group using the intention-to-treat protocol.

At 6 months, 62% (63/101) of the text message group and 62% (59/95) of the control group had completed their follow-up schedules. There were no significant differences in the attrition rates between the 2 groups. When baseline characteristics of study completers (n=122) were compared with those of noncompleters (n=74), there were no significant differences except for obesity-related quality-of-life scales. Study completers had lower obesity-related quality-of-life scale scores with mean of 24.30 (SD 10.87) versus those of noncompleters (mean 27.55, SD 7.29).

### Baseline Characteristics

There were no significant differences in sociodemographic variables between the randomized groups as shown in Table 2. Mean age was 41.02 years (SD 6.82) in the intervention group and 41.55 years (SD 6.98) in the control group. In the intervention group, mean body weight and BMI were 83.24 kg (SD 8.75) and 28.00 kg/m^2^ (SD 2.15), respectively; in the control group, mean body weight and BMI were 81.89 kg (SD 9.29) and 27.61 kg/m^2^ (SD 2.45), respectively. Half of the study participants (50.0%, 98/196) were making their first attempt at weight reduction, and more than one-third (44.4%, 87/196) were categorized as having moderate to high physical activity. Nearly 40% (78/196) of the study participants self-reported as rapid eaters and the most common comorbidity was hyperlipidemia (28.6%, 56/196).

**Table 2:** Baseline characteristics of the participants in each group (N=196).

Variables		Text message group (n=101)	Control group (n=95)	*P* ^a^
Age (years), mean (SD)		41.02 (6.82)	41.55 (6.98)	.59
Body mass index (kg/m^2^), mean (SD)		28.00 (2.15)	27.61 (2.45)	.25
Weight, mean (SD), kg		83.24 (8.75)	81.89 (9.29)	.30
Percent body fat (%), mean (SD)		25.23 (3.67)	25.75 (3.45)	.31
Systolic blood pressure (mm Hg), mean (SD)		125.62 (14.74)	123.27 (13.45)	.25
Diastolic blood pressure (mm Hg), mean (SD),		78.97 (10.38)	77.14 (9.98)	.21
Fasting glucose (mg/dL), mean (SD)		96.33 (17.06)	96.19 (14.51)	.95
Triglycerides (mg/dL), mean (SD)		169.10 (94.83)	175.17(98.06)	.66
**First attempt at weight loss, n (%)**		52 (51)	46 (48)	.77
	Current smoker, n (%)	32 (32)	26 (27)	.61
	At-risk drinking, n (%)^b^	31 (31)	28 (29)	.97
**Physical activity, n (%)** ^c^				
	Moderate	17 (17)	26 (27)	.19
	High	25 (25)	19 (20)	
**Eating pattern**				
	Rapid eating	41 (41)	41 (43)	.83
	Irregular eating	7 (7)	9 (9)	.69
	Emotional eating	4 (4)	6 (6)	.67
Married, n (%)		84 (83)	77 (81)	.84
Obesity-related QOL scales,^d^ mean (SD)		26.21 (8. 78)	25.57 (10.08)	.74
**Comorbidities, n (%)**				
	Hypertension	15 (15)	12 (13)	.81
	Diabetes	6 (6)	4 (4)	.82
	Hyperlipidemia	26 (26)	30 (32)	.45

^a^ *P* value was calculated by the *t* test for continuous variables and the chi-square test for categorical variables.

^b^ At-risk drinking was defined by average alcohol intake more than 35 g/day.

^c^ Physical activity assessed by IPAQ-SF (International Physical Activity Questionnaire-Short Form) and categorized.

^d^ Korean version of obesity-related quality-of-life (QOL) scales.

### Weight Changes

**Figure 3:**
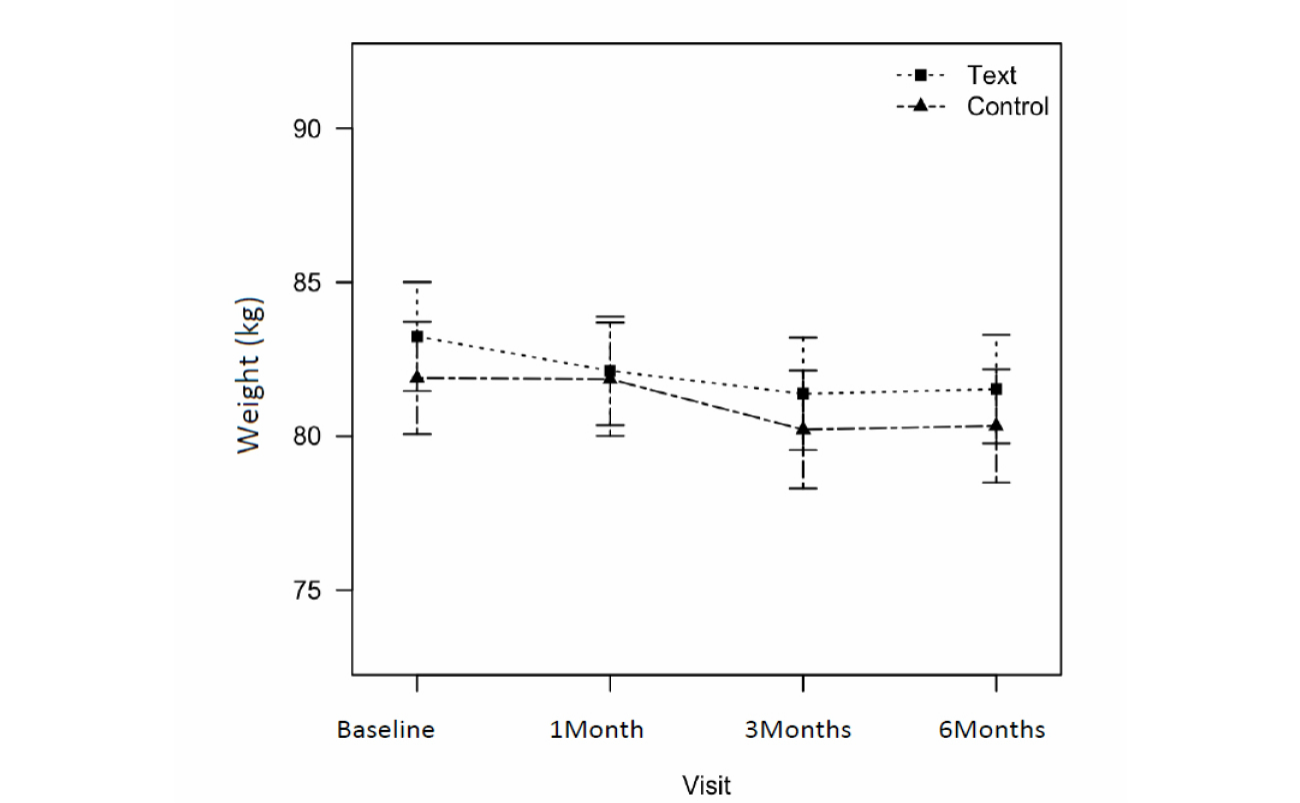
Changes in body weight between text message group and control group from baseline to 6 months.

**Figure 4:**
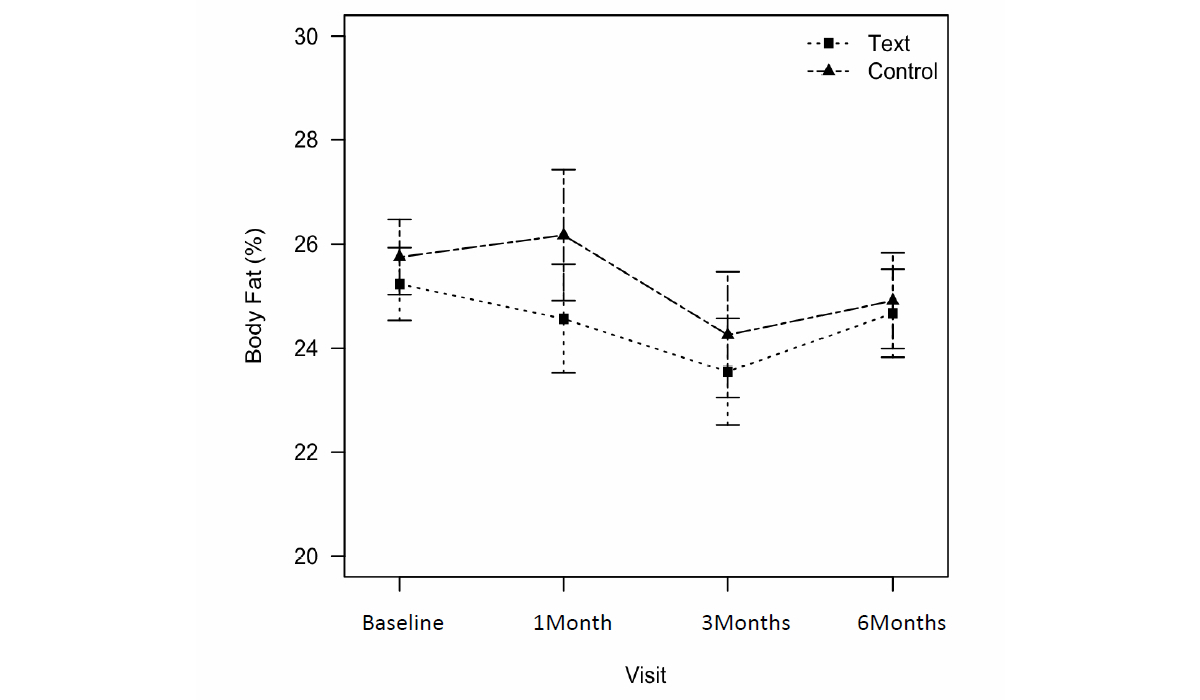
Changes in percent body fat between text message group and control group from baseline to 6 months.

Table 3 summarizes the mean body weight changes and percent body fat, physical activity, and obesity-related quality-of-life scores for the text message (n=101) and control groups (n=95) using intention-to-treat analysis. Both groups significantly reduced their body weights compared with baseline. The text message group lost a mean 1.71 kg (95% CI –2.53 to –0.88) and the control group lost a mean 1.56 kg (95% CI –2.45 to –0.66) at 6 months (Figure 3). The difference in weight loss between the 2 groups was –1.07 kg (95% CI –1.85 to –0.30) at 1 month, which was statistically significant (*P*=0.01). But, at the end of study, the difference in weight loss was –0.40 kg (95% CI –1.09 to 0.29), which was not significant. Percent body fat decreased at 3 months in both the text message (mean difference –1.68, 95% CI –2.60 to –0.79) and control (mean difference –1.29, 95% CI –2.61 to –0.37) groups but there was no significant difference between the groups (Figure 4).

**Table 3:** Baseline and changes in weight, percent body fat, physical activity, and obesity-related quality-of-life scores at each assessment point using missing data^a^ method.

Variable		Text message group (95% CI)	Within-group difference (95% CI)	*P*	Control group (95% CI)	Within-group difference (95% CI)	*P*		Between-group difference (95% CI)	*P*	
**Weight**											
	Baseline	83.24 (81.46, 85.02)	ref		81.89 (80.06, 83.73)	ref				
	1 month	82.12 (80.35, 83.89)	–1.11 (–1.62, –0.60)	<.001	81.85 (80.00, 83.70)	–0.04 (–0.62, 0.54)	.89	–1.07 (–1.85, –0.30)		.01
	3 months	81.38 (79.54, 83.21)	–1.86 (–2.48, –1.24)	<.01	80.21 (78.29, 82.14)	–1.67 (–2.42, –0.93)	<.01	–0.18 (–1.15, 0.79)		.85
	6 months	81.53 (79.75, 83.31)	–1.71 (–2.53, –0.88)	<.01	80.33 (78.48, 82.18)	–1.56 (–2.45, –0.66)	<.001	–0.15 (–1.36, 1.07)		.78
**Percent body fat**											
	Baseline	25.23 (24.53, 25.93)	ref		25.75 (25.03, 26.48)	ref				
	1 month	24.57 (23.51, 25.62)	–0.67 (–1.62, 0.28)	.17	26.17 (24.91, 27.48)	0.42 (–0.75, 1.59)	.48	–1.09 (–2.60, 0.43)		.16
	3 months	23.55 (22.51, 24.58)	–1.68 (–2.60, –0.76)	<.01	24.26 (23.04, 25.48)	–1.29 (–2.61, –0.37)	<.009	–0.19 (–1.64, 1.25)		.80
	6 months	24.67 (23.82, 25.52)	–0.56 (–1.24, 0.13)	.11	24.91 (23.99, 25.84)	–0.83 (–1.60, –0.07)	.03	0.28 (–0.75, 1.31)		.60
**Physical activity level (MET-minutes/week)**											
	Baseline	1569.6 (1161.1, 1978.1)	ref		1778.1 (1350.9, 2205.3)	ref				
	1 month	1547.3 (1053.1, 2041.4)	–22.4 (–501.8, 457.1)	.93	1807.5 (1241.6, 2373.4)	29.4 (–515.1, 573.9)	.92	–51.8 (–777.3, 673.7)		.89
	3 months	2103.3 (1620.9, 2585.6)	533.6 (47.9, 1019.3)	.03	1830.7 (1243.9, 2417.5)	52.6 (–537.5, 642.8)	.86	481.0 (–283.3, 1245.3)		.22
	6 months	2261.6 (1757.6, 2765.7)	692.0 (185.7, 1198.2)	<.008	1889.7 (1336.2, 2443.1)	111.6 (–454.7, 677.9)	.70	580.4 (–179.2, 1340.0)		.14
**Obesity-related QOL** ^b^											
	Baseline	26.85 (24.18, 29.52)	ref		25.61 (23.32, 27.90)	ref				
	6 months	27.07 (24.65, 29.50)	0.92 (–1.79, 3.63)	.51	26.2 (23.97, 28.34)	1.24 (–1.71, 4.19)	.41	–0.15 (–1.36, 1.07)		.78

^a^ Linear mixed model was used.

^b^ Korean versions of obesity-related quality-of-life (QOL) scales.

**Figure 5:**
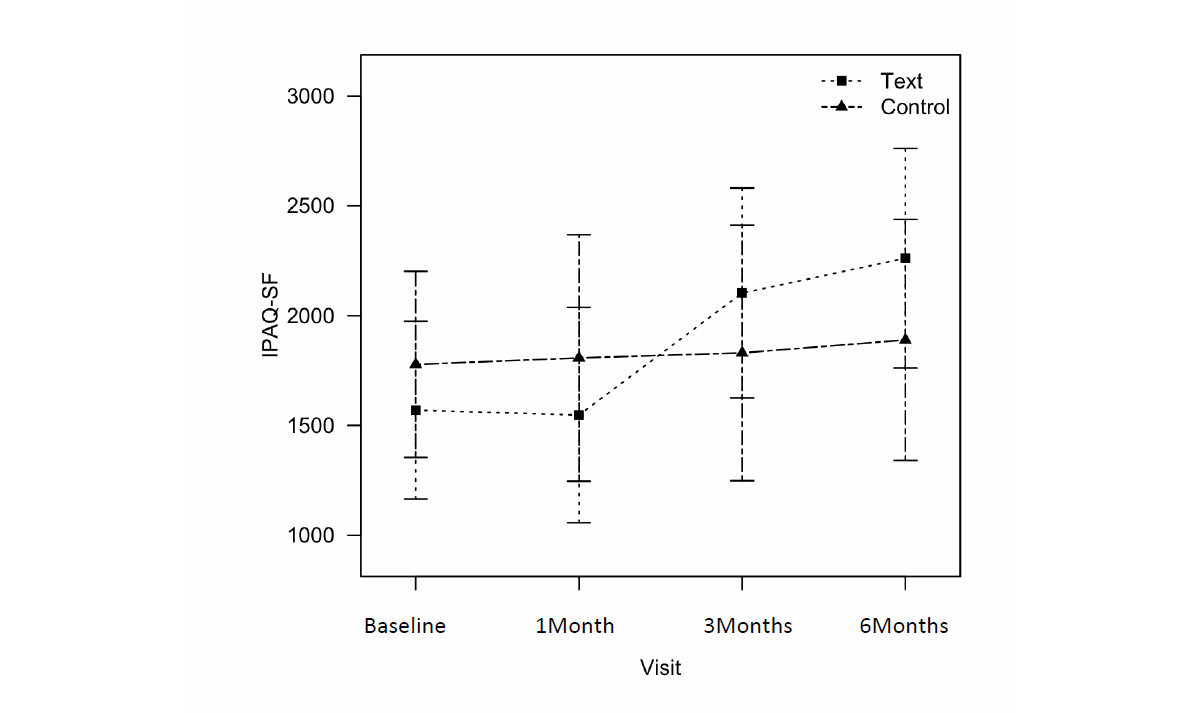
Changes in physical activities assessed by International Physical Activity Questionnaires Short Form (IPAQ-SF) between text message group and control group from baseline to 6 months.

Physical activity significantly increased in the text message group by a mean 533.6 metabolic equivalent (MET)-minutes per week at 3 months (95% CI 47.9-1019.3) and 692.0 6 MET-minutes per week at 6 months (95% CI 185.7-1198.2) (Figure 5), but no significant differences were observed between the groups. There was no change in obesity-related quality-of-life scores in either group.

### Satisfaction With and Acceptance of Text Messages

In the text-messaging intervention group, approximately two-thirds of the study participants showed positive responses to the text messages, finding them helpful (60%, 34/57), convenient (65%, 37/57), and reliable (75%, 43/57) at the 6-month evaluation (Table 4). However, the rates of positive responses for the personalized contents (39%, 22/57) and for recommending the text service to other people (46%, 26/57) were less than 50%. Still, satisfaction with the text-messaging service (63%, 36/57) and intent to use it again (63%, 36/57) was mostly positive.

**Table 4:** Satisfaction with and acceptability of the text message service (n=57).

Items	Positive response, n (%)
It helped with my weight reduction	34 (60)
It provided personalized messages	22 (39)
It was a convenient method	37 (65)
The contents were reliable	43 (75)
I was satisfied with the service	36 (63)
I would recommend it to my family or friends	26 (46)
I will use it again	36 (63)

## Discussion

### Principal Results

To the best of our knowledge, this was the first study to evaluate text messaging in a worksite weight reduction program in obese male participants. In contrast to the many positive results reported in previous trials of text-messaging interventions, our results did not indicate any significant weight reduction compared with the control group despite an overall positive perception of text messaging by the participants. At the 1-month follow-up of the study, the text messaging group reduced their weight more significantly than the control group by a difference of 1.07 kg, but at the end of the study the weight reduction difference did not show any significant change. Because both groups significantly reduced their weight by an average of 1.71 kg in the text message group and 1.56 kg in the control group compared with their baseline weight, identifying a statistically significant benefit of text messaging was more difficult to demonstrate in this cohort. In addition, study participants increased their physical activity levels and showed decreased percent body fat, but without significant differences between the groups. These surprising findings in the control group might reflect the effectiveness of the health examinations and educational sessions on weight management in the work environment.

Clinically, weight loss of 5% to 10% of initial weight are known to reduce cardiovascular disease risk factors, prevent the development of type 2 diabetes mellitus, and improve other health consequences of obesity [27,28]. At the end of the 6-month study, the text-messaging group lost 2% of their baseline weight and the control group lost 1.9% of their baseline weight. This would be considered a meaningful first step toward clinically significant weight loss.

Worksite weight management [5] is crucial in that it can provide awareness and a more structured approach for employees to address weight management. However, what is required to develop a successful program is not yet fully known [29], although the incorporation of some elements are considered essential: health education, supportive social and physical environments, and screenings followed by counseling and necessary follow-up with medical services. Employers must consider the most appropriate allocation of human, financial, and administrative resources in worksite health promotion programs to optimize their cost-effectiveness. In this regard, text messaging by mobile phone has a number of merits. Mobile phones with text messaging have a high penetration rate in nearly every society, regardless of age, socioeconomic status, and culture. Moreover, texting is fast, convenient, easy to use, broadly scalable, and can give users immediate feedback.

However, there are several core components for successful text message programs for weight loss that were lacking in our trial. For example, regular self-monitoring of physical activities, dietary intake, or tracking weight, which arise from self-determination theory [30] and have been shown to play an important role in weight loss, were not applied in our program. Also, we did not have interactive components, such as feedback or social support from peer group, based on social cognitive theory [31]. If these systems of feedback were dependent on manual input by health care workers, additional resources such as time, persons, and costs should be considered. On the other hand, if feedback systems are automated, a technique that was adopted in a number of clinical trials [32,33], after an early investment in the text message content, a messaging infrastructure could be built with adaptive flexibility.

In our program, the main function of the text messaging was to provide education and motivation in the form of personalized reminders so that the service could help participants make changes in lifestyle. Systemic review [34] regarding periodic messaging intervention on health behaviors also showed that periodic messaging interventions yield positive results for short-term health behavior changes. But the review also suggested the provision of feedback and specific strategies appeared to be important for the success. We did not provide participants with bidirectional communication capabilities or self-monitoring functions. This kind of additional functionality might have increased the effectiveness of our text messaging intervention, increasing the likelihood of finding a significant improvement compared with the control group.

In terms of adherence to study, obesity-related quality-of-life scales were significantly lower in the study completers than in noncompleters, but these scales were originally developed to measure various quality of life in obese patients whose mean BMI was more than 30 and diverse age and gender group. Our study participants were relatively mildly obese as classified by BMI and primarily an active working male population in their forties. So it is possible that the Korean version of the quality-of-life scales did not reflect quality of life in our study participants very well.

### Comparison With Prior Work

According to a recent meta-analysis [35] regarding text messages for weight management, there were several elements of behavior modification as well as function of text messaging in qualitative analysis of 13 trials. Among elements of behavior modification, all messages contained nutritional information and exercise was the second most common target behavior (92%, 12/13). Social support with nutritional education was used in more than half of trials (54%, 7/13). Our study also used physical activities with tailored nutritional tips for the contents of text message, but social support was not part of the program.

In function of text-messaging formats, more than half of trials (54%, 7/13) used text messaging to report self-monitoring data and to provide tailored feedback responses to participants around food intake, physical activity levels, and weight. Approximately one-third of trials (31%, 4/13) adopted group sessions, and one-quarter of trials (23%, 3/13) employed individual consultation or telephone coaching. In our trial, monitoring, tailored feedback, or telephone coaching were not provided, but group sessions for education and individual care by nurses on request were given during study period. In terms of self-regulation theory-driven health behavior change [36], self-monitoring with immediate feedback is especially important in making text messaging successful in weight reduction. Recent clinical trials with minority women [37] and in Beijing [38] used text messaging as a reminder and monitoring tool for predetermined behavior goals, and showed positive outcomes in weight reduction.

### Limitations and Strengths

There could be multiple reasons that text messaging did not prove its effectiveness in our study. First, our study participants were recruited on the recommendation of managing nurses based on routine health examinations, whereas most other trials recruited from volunteers; thus, there may be differences between our participants and volunteers. Second, we attempted individual randomization within organizations, which could have led to contamination between the text messaging and control groups.

Third, the most successful text-messaging program participants in prior studies have been women, in contrast to our male participants, and in our study the retention rate was approximately 60%, lower than the 90% that has been reported for female participants [37]. Fourth, in our survey regarding satisfaction and acceptance, most participants in the intervention group did not feel that they had received personalized messages, although we tried to personalize the messages based on each participant’s health behavior and cardiovascular and metabolic profiles. The absence of the self-monitoring with feedback functionality in the text messaging reduced the potential desirable effect on the subjective recognition of personalized contents and also on weight management.

In spite of the preceding limitations, our study had multiple strengths. First, we evaluated the text-messaging reminder function in a randomized clinical trial, which alone did not appear to have an effect on weight reduction. Second, with our focus on male participants, our study adds to the limited evidence base of possible interventions for obese males. Third, we attempted to use text messaging in work environments, which could have great impact on individual health behavior changes with proper implementation. Future studies that focus on text messages with self-monitoring and feedback in the workplace setting are needed.

### Conclusions

In a 6-month trial, adding tailored text messages as reminders did not show any significant effects on weight reduction in obese male participants in worksite weight management programs. Further testing is needed to study the potential benefit of immediate interactive feedback and an emphasis on self-monitoring activities on its effectiveness in worksite wellness programs.

## Multimedia Appendix 1

CONSORT-EHEALTH checklist V1.6.2 [39].

## Abbreviations

BMI: body mass index
IPAQ-SF: International Physical Activity Questionnaire-Short Form
KDHC: Korea District Heating Corporation
KEC: Korea Expressway Corporation
KOGAS: Korea Gas Corporation
