# Supplementary material for: Effectiveness of 6 Months of Tailored Text Message Reminders for Obese Male Participants in a Worksite Weight Loss Program: Randomized Controlled Trial
Source: JMIR Mhealth Uhealth. 2015 Feb 3;3(1):e14. doi: 10.2196/mhealth.3949 (PMC4342743; doi:10.2196/mhealth.3949)
Supplement: Supplementary file 1 [file mhealth_v3i1e14_app1.pdf]

CONSORT-EHEALTH Checklist V1.6.2 Report

(based on CONSORT-EHEALTH V1.6), available at [http://tinyurl.com/consort-ehealth-v1-6].

3949

Date completed
10/14/2014 17:45:53
by
Ju Young Kim

Effectiveness of 6 month automatic tailored text messaging for obese male participants in worksite weight loss program: Randomized controlled trials

TITLE

1a-i) Identify the mode of delivery in the title
"automatic tailored text messaging"
1a-ii) Non-web-based components or important co-interventions in title
"in worksite weight loss program"
1a-iii) Primary condition or target group in the title
"for obese male participants in worksite weight loss program"

ABSTRACT

1b-i) Key features/functionalities/components of the intervention and comparator in the METHODS section of the ABSTRACT
"Participants were randomly allocated to one of the following two groups, lasting 24 weeks : (1) intervention group that included automatic tailored text messages given every other day and four off line education sessions about weight control during 6 months ; (2) control group that received four off line education sessions about weight control during 6 months "
1b-ii) Level of human involvement in the METHODS section of the ABSTRACT
"included automatic tailored text messages given every other day and four off line education"
1b-iii) Open vs. closed, web-based (self-assessment) vs. face-to-face assessments in the METHODS section of the ABSTRACT
"Men with body mass index (BMI, kg/m2) more than 25 were recruited from Korea district heating corporation, Korea expressway corporation and Korea gas corporation recommended by healthcare managing nurses."
1b-iv) RESULTS section in abstract must contain use data
"A total of 205 obese men were randomized into intervention group (n=104) or control group (n=101). At the end of 6 months, the intervention group (n=63) lost 1.7 kg (SE 0.4) and control group (n=59) lost 1.6 kg (SE 0.5) "
1b-v) CONCLUSIONS/DISCUSSION in abstract for negative trials
"Automatic tailored text messages did not take significant effects on weight loss in obese men. Lack of interactive tools such as social network services with tailored feedbacks may be the main cause of negative result in automatic text messaging in worksite weight loss program."

INTRODUCTION

2a-i) Problem and the type of system/solution
"Usually development and implementation of worksite weight management program needs sufficient resources including financial, time, staff and space. Also there are reported barriers to participation[11,12] in worksite program, which includes insufficient incentives, inconvenient locations, and time limitations.
Using text messages in worksite weight management program can be a useful method to overcome with time, location and cost barriers. There are some studies using telephone coaching[13] or online based weight management[14] in worksite weight management but no studies have been reported regarding the effect of text messaging on weight loss in worksite weight management program."
2a-ii) Scientific background, rationale: What is known about the (type of) system
"Use of text messaging in health behavior change[8-10] has been more and more adopted not only because of wide availability, inexpensiveness, instant effects on users but also of its effectiveness in some health behavior changes."

METHODS

3a) CONSORT: Description of trial design (such as parallel, factorial) including allocation ratio
"So we conducted a 6 month randomized controlled trial if using automatic tailored text message service would be a more effective way of losing weight loss compared with standard care in worksite weight management program."
3b) CONSORT: Important changes to methods after trial commencement (such as eligibility criteria), with reasons
"No methodological changes were done during the study period. "
3b-i) Bug fixes, Downtimes, Content Changes

4a) CONSORT: Eligibility criteria for participants
"Participants were recruited in 2011 from 3 public institutions, Korea Gas Corporation (KOGAS), Korea District Heating Corporation (KDHC), and Korea Expressway Corporation (KEC) with a help of healthcare managing nurses. The nurses screened potential participants for meeting the following inclusion criteria: age between 20 and 60 years old, obesity by body mass index more than 25, not taking medications known to cause weight gain, and owns a mobile phone and uses text messaging services."
4a-i) Computer / Internet literacy
We did not use the computer or internet.
4a-ii) Open vs. closed, web-based vs. face-to-face assessments:
"Participants who are eligible for this study were invited to participate in this program by the study coordinator after finishing their annual health examination in a hospital. "
4a-iii) Information giving during recruitment
"Participants who are eligible for this study were invited to participate in this program by the study coordinator after finishing their annual health examination in a hospital. "
4b) CONSORT: Settings and locations where the data were collected
"Participants were recruited in 2011 from 3 public institutions, Korea Gas Corporation (KOGAS), Korea District Heating Corporation (KDHC), and Korea Expressway Corporation (KEC) with a help of healthcare managing nurses.....Participants who are eligible for this study were invited to participate in this program by the study coordinator after finishing their annual health examination in a hospital. ... The study took place between May 2011 to December 2011 in Seongnam, Korea."
4b-i) Report if outcomes were (self-)assessed through online questionnaires
"Research group visited worksite and provided group educational sessions with printed materials for management of obesity and feedback by self - reported questionnaires at baseline, 1 month, 3 month and 6 month. Printed materials were different in every educational session according to the topic issued. Nurses were checking weight, brief counseling about weight loss tips and encouraging participation in this program every month."

4b-ii) Report how institutional affiliations are displayed
"Employees working in public institution annually underwent standardized medical examinations in local cooperative hospitals....There are nurses who take care of worksite disease prevention with health promotion program in public institution.
Participants were recruited in 2011 from 3 public institutions, Korea Gas Corporation (KOGAS), Korea District Heating Corporation (KDHC), and Korea Expressway Corporation (KEC) with a help of healthcare managing nurses. "
5) CONSORT: Describe the interventions for each group with sufficient details to allow replication, including how and when they were actually administered
5-i) Mention names, credential, affiliations of the developers, sponsors, and owners
"We used commercial automatic text message senders (®Munjanara, http://www.munjanara.co.kr/, Seoul, Korea) for tailored messages and we could not have interactive feedback program nor showing the cumulative track of weight. "

5-ii) Describe the history/development process
"Contents of text messages were developed to be both automatic and personally tailored messages according to their lifestyle and metabolic risk factors. So text messages were unidirectional. Three family physicians, one psychiatrist and two dietitians got together to develop contents of text messages regarding motivation, nutritional tips, helpful recipes in their own risk factors and exercise tips with motivation. Text messages were given to intervention group three times a week at morning and consisted of “Goal setting and behavior change”, “Education and tips for nutrition”, and “Exercise and get more active” themes.
“Goal and behavior change” part included goal setting, self- monitoring, needs for weight control, overcoming barriers and stop emotional eating.
“Education and tips for nutrition” part included meal replacement or substitution, meal planning, tips for eating out, ideal macronutrient compositions, education about empty calories and also recipes for lowering triglyceride level or uric acid or fasting glucose level tailored to participant’s profiles.
“Exercise and get more active” included tips for increasing non exercise physical activity level, education of type, duration and frequency in exercises, and showing calories burn per exercise type per hour. Examples of contents of text messages are shown at table 1. "
5-iii) Revisions and updating
"No methodological changes were done during the study period. "
5-iv) Quality assurance methods
"The study evaluated a text message based application according to eating behaviors and physical activity levels by questionnaires and metabolic risk profile assessed by laboratory examinations and anthropometric measurements. Eating patterns and amounts were assessed by 24 hour dietary recall analysis and eating behaviors such as emotional eating, social influences, convenience food and alcohol consumption by frequency and amount were also assessed. Physical activity level was assessed and categorized by International Physical Activity Questionnaire- Short Form[15] (IPAQ-SF).
Fasting glucose level, uric acid, serum triglyceride level were categorized for development of tailored text messages. "
5-v) Ensure replicability by publishing the source code, and/or providing screenshots/screen-capture video, and/or providing flowcharts of the algorithms used
"Text messages were given to intervention group three times a week at morning and consisted of “Goal setting and behavior change”, “Education and tips for nutrition”, and “Exercise and get more active” themes.
“Goal and behavior change” part included goal setting, self- monitoring, needs for weight control, overcoming barriers and stop emotional eating.
“Education and tips for nutrition” part included meal replacement or substitution, meal planning, tips for eating out, ideal macronutrient compositions, education about empty calories and also recipes for lowering triglyceride level or uric acid or fasting glucose level tailored to participant’s profiles.
“Exercise and get more active” included tips for increasing non exercise physical activity level, education of type, duration and frequency in exercises, and showing calories burn per exercise type per hour. Examples of contents of text messages are shown at table 1."
5-vi) Digital preservation
We did not apply internet program nor digital preservation.
5-vii) Access
We did not apply the internet or web based education, It was only text messages by cell phone or printed materials.
5-viii) Mode of delivery, features/functionalities/components of the intervention and comparator, and the theoretical framework
"Contents of text messages were developed to be both automatic and personally tailored messages according to their lifestyle and metabolic risk factors. So text messages were unidirectional. Three family physicians, one psychiatrist and two dietitians got together to develop contents of text messages regarding motivation, nutritional tips, helpful recipes in their own risk factors and exercise tips with motivation. Text messages were given to intervention group three times a week at morning and consisted of “Goal setting and behavior change”, “Education and tips for nutrition”, and “Exercise and get more active” themes.
“Goal and behavior change” part included goal setting, self- monitoring, needs for weight control, overcoming barriers and stop emotional eating.
“Education and tips for nutrition” part included meal replacement or substitution, meal planning, tips for eating out, ideal macronutrient compositions, education about empty calories and also recipes for lowering triglyceride level or uric acid or fasting glucose level tailored to participant’s profiles.
“Exercise and get more active” included tips for increasing non exercise physical activity level, education of type, duration and frequency in exercises, and showing calories burn per exercise type per hour."
5-ix) Describe use parameters
"Text messages were given to intervention group three times a week at morning and consisted of “Goal setting and behavior change”, “Education and tips for nutrition”, and “Exercise and get more active” themes."
5-x) Clarify the level of human involvement
"Research group visited worksite and provided group educational sessions with printed materials for management of obesity and feedback by self - reported questionnaires at baseline, 1 month, 3 month and 6 month. Printed materials were different in every educational session according to the topic issued. Nurses were checking weight, brief counseling about weight loss tips and encouraging participation in this program every month"
5-xi) Report any prompts/reminders used
Text messages as reminder function was main delivery tool in our study. And any other reminder or prompts were not used in the study.
5-xii) Describe any co-interventions (incl. training/support)
Training was not needed in our study, because our study was usually education with receiving a text message without feedback.
6a) CONSORT: Completely defined pre-specified primary and secondary outcome measures, including how and when they were assessed
"Weight change was primary main outcome and it was also evaluated with percent body fat at baseline, 1month, 3month and 6 month measured by nurses using a bioelectrical impedance analysis (InBody U20®, Seoul, Korea)."

6a-i) Online questionnaires: describe if they were validated for online use and apply CHERRIES items to describe how the questionnaires were designed/deployed
We did not use online questionnaires.
6a-ii) Describe whether and how “use” (including intensity of use/dosage) was defined/measured/monitored
In our program text message was functioning as reminder, so we could not evaluate the usage pattern of text message.
6a-iii) Describe whether, how, and when qualitative feedback from participants was obtained

"Satisfaction and acceptance of this text message program in intervention group was evaluated at 6 month by rating the program overall (e.g. Were you satisfied with the text message program? How much help did you get for text messages in weight management?) with 5 point Likert rating scales (Strongly agree – strongly disagree). Positive response was regarded as being somewhat agree to strongly agree level. "

6b) CONSORT: Any changes to trial outcomes after the trial commenced, with reasons

"No methodological changes were done during the study period. "

7a) CONSORT: How sample size was determined

7a-i) Describe whether and how expected attrition was taken into account when calculating the sample size

"A sample size calculation by prior study[18] suggested that total of 124 participants provided a power of 0.8 to detect a group difference at 6 month with alpha=0.05. Allowing for the attrition rate as 30%, total of 207 participants were needed to perform this study. "

7b) CONSORT: When applicable, explanation of any interim analyses and stopping guidelines

We did not conduct the interim analysis or stopping guidelines.

8a) CONSORT: Method used to generate the random allocation sequence

"Random allocation was performed by blocked randomization methods using web based randomization in Medical Research Collaborating Center of Seoul National University Hospital."

8b) CONSORT: Type of randomisation; details of any restriction (such as blocking and block size)

"Random allocation was performed by blocked randomization methods with blocked size of 4 and 6 using web based randomization in Medical Research Collaborating Center of Seoul National University Hospital."

9) CONSORT: Mechanism used to implement the random allocation sequence (such as sequentially numbered containers), describing any steps taken to conceal the sequence until interventions were assigned

"Random allocation was performed by blocked randomization methods using web based randomization in Medical Research Collaborating Center of Seoul National University Hospital. A trained research assistant who did not participate in running educational session or recruiting with potential participant was scheduling and sending text messages to intervention group by commercial software automatic text message senders. "

10) CONSORT: Who generated the random allocation sequence, who enrolled participants, and who assigned participants to interventions

"Participants who are eligible for this study were invited to participate in this program by the study coordinator after finishing their annual health examination in a hospital. Evaluation of text messaging program was performed by unblinded randomized controlled trial in 1:1 to intervention or to comparison group. Random allocation was performed by blocked randomization methods using web based randomization in Medical Research Collaborating Center of Seoul National University Hospital. A trained research assistant who did not participate in running educational session or recruiting with potential participant was scheduling and sending text messages to intervention group by commercial software automatic text message senders. No methodological changes were done during the study period. "

11a) CONSORT: Blinding - If done, who was blinded after assignment to interventions (for example, participants, care providers, those assessing outcomes) and how

11a-i) Specify who was blinded, and who wasn't

"Evaluation of text messaging program was performed by unblinded randomized controlled trial in 1:1 to intervention or to comparison group."

11a-ii) Discuss e.g., whether participants knew which intervention was the "intervention of interest" and which one was the "comparator"

"Participants were explained about text messaging intervention in work site weight reduction education program."

11b) CONSORT: If relevant, description of the similarity of interventions

"Comparison group received the same group educational sessions with printed materials at baseline, 1month, 3month and 6 month and checked weight with brief counseling about diet, exercise, coping with emotional eating, tips for avoiding overeatings, weight maintenance strategies with nurses in their institution. The comparison group did not receive automatic tailored text message services. "

12a) CONSORT: Statistical methods used to compare groups for primary and secondary outcomes

"Effect of intervention via text messaging on weight changes were analyzed using a linear mixed effects model for repeated measures[19], which uses all available data and provides valid results in the presence of missing data are missing at random. The model considered the age, treatment group, time and group by time interaction as fixed effect and incorporated random effects for individual subjects, such as a random intercept and a random slope (with respect to time)."

12a-i) Imputation techniques to deal with attrition / missing values

". Effect of intervention via text messaging on weight changes were analyzed using a linear mixed effects model for repeated measures[19], which uses all available data and provides valid results in the presence of missing data are missing at random. The model considered the age, treatment group, time and group by time interaction as fixed effect and incorporated random effects for individual subjects, such as a random intercept and a random slope (with respect to time)."

12b) CONSORT: Methods for additional analyses, such as subgroup analyses and adjusted analyses

"Physical activities and scores of obesity related quality of life were also analyzed by linear mixed model."

RESULTS

13a) CONSORT: For each group, the numbers of participants who were randomly assigned, received intended treatment, and were analysed for the primary outcome

"Study enrollment and retention flow was shown as figure 1. A total of 222 individuals were eligible for participation assessed by health care managing nurses. Among those eligible, 205 were final study population and randomized to either text message group (n=104) or the control group (n=101). Baseline weight and body fat percent was measured at the time of educational session with a portable body impedance analysis, so if participant did not show up at the education session, we could not get baseline data of body weight. So we analyzed 101 of text message group and 95 of control group by intention to treat protocol.
At 6 month, 62.4% (63/101) of text message group and 62.1% (59/95) of control group completed their follow up schedule. There were no significant differences in attrition rate between both groups."

13b) CONSORT: For each group, losses and exclusions after randomisation, together with reasons

Please see the CONSORT flow diagram suggested in figure 1.

13b-i) Attrition diagram

"Figure1"

14a) CONSORT: Dates defining the periods of recruitment and follow-up

". The study took place between May 2011 to December 2011 in Seongnam, Korea. "

14a-i) Indicate if critical "secular events" fell into the study period

There are no secular events.

14b) CONSORT: Why the trial ended or was stopped (early)

The planned time was ended.

15) CONSORT: A table showing baseline demographic and clinical characteristics for each group

Table 2.

15-i) Report demographics associated with digital divide issues

Table 2.

16a) CONSORT: For each group, number of participants (denominator) included in each analysis and whether the analysis was by original assigned groups

16-i) Report multiple “denominators” and provide definitions

"There were no significant differences in sociodemographic variables between randomized groups. Mean age was 41.0 years (SD 6.8) in intervention group and 41.6 years (SD 7.0) in control group. Mean body weight and body mass index was 83.2 kg (SD 8.8) , 25.2 kg/m2 (SD 3.7)in intervention group, and 81.9 kg (SD 8.8), 25.7 kg/m2 (SD 3.5)in control group. Half of study participants (49.0%, 98/196) were first attempter in weight reduction and more than one third (44.4%, 87/196) were categorized as moderate to high physical activities. Nearly 40% of study participants were responded as a rapid eater, and most common comorbidity was hyperlipidemia (28.6%, 56/196). "

16-ii) Primary analysis should be intent-to-treat

"Table 3 summarized the mean body weight changes as well as percent body fat, physical activities and obesity related quality of life scales in text message group and control group. Both group significantly reduced body weight compared with baseline. Text message group lost an average of 1.6 kg (SD 8.1) and control group also lost an average of 1.6kg (SD 7.0) at 6 month as presented in figure2,

17a) CONSORT: For each primary and secondary outcome, results for each group, and the estimated effect size and its precision (such as 95% confidence interval)

"Table 3 summarized the mean body weight changes as well as percent body fat, physical activities and obesity related quality of life scales in text message group (n=101) and control group (n=95) by intention to treat analysis. Both group significantly reduced body weight compared with baseline. Text message group lost an average of 1.6 kg (SD 8.1) and control group also lost an average of 1.6kg (SD 7.0) at 6 month as presented in figure2, but no significant difference between the group. Physical activities were significantly increased in text message group by an average of 533.6 MET-minutes per week at 3 month and 692.0 6 MET-minutes per week at 6 month (figure 3), but no significant changes were observed between the group. Percent body fat was decreased at 3 month in both group (figure 4), but there was no significant difference between the group and did not correlated with reported physical activities.
Obesity related quality of life scales were not changed in both groups."

17a-i) Presentation of process outcomes such as metrics of use and intensity of use

We did not evaluate the process outcomes.

17b) CONSORT: For binary outcomes, presentation of both absolute and relative effect sizes is recommended

We did not evaluate the binary outcome.

18) CONSORT: Results of any other analyses performed, including subgroup analyses and adjusted analyses, distinguishing pre-specified from exploratory

We did not conduct the subgroup analysis.

18-i) Subgroup analysis of comparing only users

We did not perform subgroup analysis of only users.

19) CONSORT: All important harms or unintended effects in each group

We did not have unintended effects or important harms reported.

19-i) Include privacy breaches, technical problems

These were not reported and we did not have technical problems.

19-ii) Include qualitative feedback from participants or observations from staff/researchers

"In text messaging intervention group, about two thirds of study participants showed positive response to text message as being helpful (59.6%, 34/57), convenient method (64.9%, 37/57), and reliable (75.4%, 43/57) at 6 month evaluation. But rate of positive response for being personalized contents (38.6%, 22/57) or recommendation to other people (45.6%, 26/57) was below 50%. Still satisfaction of the text messaging program (63.1%, 36/57) and intent to reuse the program (63.1%, 36/57) showed somewhat high positive response rate. "

DISCUSSION

20) CONSORT: Trial limitations, addressing sources of potential bias, imprecision, multiplicity of analyses

20-i) Typical limitations in ehealth trials

"There could be several reasons text messaging did not prove its effectiveness in our study.
First, our study participants were recruited by recommendation of health managing nurses based on routine health examination, where most other trials recruited from volunteers for using text messaging. So it may lead the difference from volunteer group.
Second, we tried individual based randomization in organization, which could lead to contamination between text messaging group and control group. Third, most of successful text messaging program participants were women, quite different in our male participants. And in our study retention rate was about 60%, lower than that of female participants, which was reported 90%[25]. Fourth, in our survey regarding satisfaction and acceptance, most participants in intervention group did not feel they had personalized messages, though we tried to make messages personalized according to participant’s health behavior, cardiovascular and metabolic profiles. Without implementing the self monitoring with feedback function in text messaging, it would not take a desirable effect on subjective feeling of personalized contents and also on weight management."

21) CONSORT: Generalisability (external validity, applicability) of the trial findings

21-i) Generalizability to other populations

"we also evaluated its usefulness in male participants, and added some evidence in considering intervention in obese male participants. Third, we tried text messaging in workplace environments, which could have a big impact on person’s health behavior change with proper implementation. "

21-ii) Discuss if there were elements in the RCT that would be different in a routine application setting

"In our program, main function of text messaging was intended to have educational role as a personalized reminder, so that it could help participants making changes in their lifestyle. But we did not provide participants with the automatic feedback or self monitoring functions. That might be one of reasons why our automatic reminder text messaging did not gain significant effect compared with control group, though we showed nonsignificant weight reduction results in text messaging group. "

22) CONSORT: Interpretation consistent with results, balancing benefits and harms, and considering other relevant evidence

22-i) Restate study questions and summarize the answers suggested by the data, starting with primary outcomes and process outcomes (use)

"So far as we know, this was the first study evaluating text messaging in worksite weight reduction program in male obese participants. Contrary to many positive results in previous trials of text messaging intervention, our results did not prove its effectiveness significantly in weight reduction compared to control group. But both groups significantly reduced their weight by an average of 1.7 kg in text message group, 1.6kg in control group compared to their baseline weight. Also our study participants increased physical activities with decreased percent body fat within group, though did not show significant difference between the group. It might reflect the fact that health examination with providing educational session had effect on weight management in worksite environment."

22-ii) Highlight unanswered new questions, suggest future research

". First, we first evaluated the reminder function of text messaging in randomized clinical trial, which by itself did not seem to have effect on weight reduction. Second, we also evaluated its usefulness in male participants, and added some evidence in considering intervention in obese male participants. Third, we tried text messaging in workplace environments, which could have a big impact on person's health behavior change with proper implementation.
Future studies focusing on text messages with self monitoring and feedback function in workplace setting will be needed."

Other information

23) CONSORT: Registration number and name of trial registry

Trial Registration: ISRCTN 39629189

24) CONSORT: Where the full trial protocol can be accessed, if available

We did not submit our full trial protocol.

25) CONSORT: Sources of funding and other support (such as supply of drugs), role of funders

"This study was supported by grant no 11-2010-028 from the SNUBH Research Fund. "

X26-i) Comment on ethics committee approval

"The Institution Review Board of Seoul National University Bundang Hospital approved the study. (IRB number B-0908-082-014). "

x26-ii) Outline informed consent procedures

"Participants who are eligible for this study were invited to participate in this program by the study coordinator after finishing their annual health examination in a hospital. Participants were explained about text messaging intervention in work site weight reduction education program and gave written informed consents about using their hospital information in text messages."

X26-iii) Safety and security procedures

"Participants were asked to contact the researchers by telephone or email if they had questions regarding the trial."

X27-i) State the relation of the study team towards the system being evaluated

"None declared. "
